# Dietary Resin Acid Concentrate Improved Performance of Broiler Chickens and Litter Quality in Three Experiments

**DOI:** 10.3390/ani11113045

**Published:** 2021-10-25

**Authors:** Krzysztof Lipiński, Juhani Vuorenmaa, Magdalena Mazur-Kuśnirek, Katarzyna Sartowska-Żygowska, Hannele Kettunen

**Affiliations:** 1 Department of Animal Nutrition and Feed Science, University of Warmia and Mazury in Olsztyn, 10-718 Olsztyn, Poland; krzysztof.lipinski@uwm.edu.pl (K.L.); magdalena.mazur@uwm.edu.pl (M.M.-K.); 2 Hankkija Ltd., FI-05801 Hyvinkää, Finland; juhani.vuorenmaa@hankkija.fi; 3 NOACK Polen Sp. z o.o., ul. Poloneza 93, 02-826 Warsaw, Poland; ksartowska@noackgroup.com

**Keywords:** resin acid concentrate, feed additives, gut health

## Abstract

**Simple Summary:**

Resin acids are antimicrobial and anti-inflammatory compounds derived from spruce and pine trees. In a previous study, in which a mixture of resin acids and fatty acids from pine and spruce trees was added to the feeds of broiler chickens, they grew heavier, and their footpads stayed in better condition. Here, three separate trials were conducted to study whether dietary resin acids without the fatty acids have the same effects on broiler chickens. Broiler chicken diets were amended with different dosages of a resin acid concentrate product. Bird weight gain increased, and the efficiency of broiler production was improved when the resin acid concentrate was added to the diet at 125–250 g/ton. The litter material of pens stayed drier in resin acid concentrate groups, which may indicate better intestinal condition of the birds. However, the footpad condition was unaffected by the experimental diets. The results suggest that the resin acid concentrate improves bird weight gain and the efficiency of broiler production. Therefore, the resin acid concentrate may be a promising feed additive for broiler chickens.

**Abstract:**

Dietary coniferous resin acids have previously been suggested to support the intestinal integrity of broiler chickens by reducing mucosal collagen degradation. The present study examined the effects of resin acid concentrate (RAC) on broiler performance and litter quality. In trial 1, RAC was added to diets at 0, 125, 250, or 1250 g/ton, while in trials 2 and 3, RAC dosing was 0 or 175 g/ton. Bird weight, feed consumption, mortality, feed conversion ratio (FCR), European Efficiency Index (EEI), litter moisture, and footpad dermatitis (FPD) lesions were measured. In trial 1, RAC at 125 and 250 g/ton improved weight gain and EEI, while RAC at 1250 g/ton group did not differ from control. Feed consumption, FCR, FPD scores and mortality were similar in all treatments, but litter quality was improved by all doses of RAC. In trials 2 and 3, RAC increased the final weight of birds, improved FCR, EEI, and litter quality, but had no effects in other parameters. In summary, RAC at 125–250 g/ton improved bird performance and thus shows promise as a feed additive. The dryer litter in RAC treatments may suggest improved intestinal condition as a response to in-feed resin acids.

## 1. Introduction

During the past two decades, novel research methodologies have provided a lot of detailed information on the association of intestinal inflammation and suboptimal production performance in poultry and other farm animals [1,2,3]. Gut leakage has even been connected to skeletal health and lameness in broiler chickens [4]. The poultry industry has responded by developing feed supplements and additives with the potential to balance intestinal microbiota and support animal performance, such as organic acids, probiotics, prebiotics, and phytobiotics [5,6,7,8].

A newcomer to the group of plant-based feed supplements is tall oil fatty acid (TOFA) [9] which contains ~90% free fatty acids and ~9% resin acids of the coniferous trees Scots pine (*Pinus sylvestris* L.) and Norway spruce (*Picea abies* L.) and which has since 2015 been marketed for farm animal feeds in some areas and countries such as the European Union, Norway, Switzerland, Turkey, Thailand, and Mexico. Amending diets with TOFA has improved the production performance of broiler chickens [9,10,11], turkeys [12], and sows [13]. In vitro, TOFA has inhibited the growth of pathogenic strains of *Clostridium perfringens* [9,11,14], while necrotic enteritis challenge trials with broilers suggest improved performance of TOFA-fed birds under intestinal challenges [9,10]. Favorable changes in intestinal microbiota [11,13] may partly explain the better production performance of TOFA-treated farm animals.

While the fatty acids of TOFA, mostly linoleic and oleic acids, are common in poultry feeds because of their presence in other feedstuffs, no history seems to exist for the intentional addition of purified coniferous resin acids to farm animal feeds. Resin acids, the main components of coniferous rosin, are hydrophobic diterpene carboxylic acids with antibacterial, antifungal, and anti-inflammatory activity [15,16,17]. An array of resin acid types is produced by coniferous trees, with abietic, dehydroabietic, pimaric, and palustric acids being among the most common types [18], and these are also found in TOFA [11]. The molecular weight of most coniferous resin acids is approximately 302.5 g/mol, and their solubility and biological properties differ somewhat according to their individual molecular characteristics [19]. The outstanding wound healing properties of rosin, known for centuries by the traditional medicine of Eurasia, are now applied also in western medicine [20].

When purified natural mixture of coniferous resin acids was fed to broiler chickens at the dietary level of 200 g/ton for three weeks, the density of inflammation-associated T-cells in the duodenal mucosa was reduced, and the collagenolytic matrix metalloproteinase activity in the ileal mucosa was inhibited [21]. These results indicated anti-inflammatory potential of ingested resin acids in the gastrointestinal tract of broiler chickens. The authors concluded that due to the inhibition of collagen degradation in the extracellular matrix of intestinal tissue, in-feed resin acids may support gut barrier integrity and intestinal homeostasis [21]. Disturbed intestinal integrity, often referred to as gut leakage, results in wet litter syndrome and poor performance of birds [22], higher incidence of footpad dermatitis (FPD) [23], and increased translocation of enteric bacteria to internal organs [24].

The hypothesis of the present study was that if resin acids have a positive effect on gut barrier and homeostasis, they may also improve the performance of broiler chickens, reduce the moisture of litter material in pens, and inhibit the development of FPD lesions. Three experiments were conducted including different doses of RAC in broiler feeds until 35 days of age. Bird performance, efficiency of broiler production, FPD incidence, and litter moisture were measured in all three trials. The dosing of RAC was from 125 to 1250 g/ton, the highest dosing giving information on the safety of the product to the birds.

## 2. Materials and Methods

### 2.1. Test Material

The product RAC (Hankkija Ltd., Hyvinkää, Finland) contained 40.0% tall oil rosin, 60% of food grade whole grain wheat flour, and antioxidants BHT (175 ppm) and BHA (175 ppm) in the form of dry flour. Tall oil rosin was derived from the tree species Scots pine (*Pinus sylvestris* L.) and Norway spruce (*Picea abies* L.), and it was produced by Forchem Ltd. (Rauma, Finland). The analyzed total content of resin acids in RAC was 37.5% (±0.2%).

### 2.2. Animals and Housing

The animals used in the present study were kept in accordance with the provisions of the Act of 15 January 2015 on the Protection of Animals Used for Scientific or Educational Purposes [25]. In all trials, the birds were housed in open pens with wood pellet bedding material, under typical living conditions (University Poultry Research Farm, 10-718 Olsztyn, Poland). Pen size was 3.22 m × 1.11 m in trial 1 and 1.61 m × 1.11 m in trials 2 and 3. The birds had an unlimited access to feed and water at all times during the experiments. Daily health, mortality, and culls were recorded, including cause of death and reason for culling. Health status was analyzed by visual inspection (general health status, morbidity, quality of faces). Mortality was recorded as it occurred, along with the bird’s body weight and cause of death, if known. Birds were checked at a minimum of twice a day, and the birds were euthanized by manual cervical dislocation if they were lame (e.g., unable to support their body weight or walk more than 1 m), unthrifty (e.g., visibly smaller than pen-mates, ruffled feathers, lethargic), or had serious injuries, such as a broken wing.

The temperature of the animal facility at the first day of life was maintained at 31–33 °C and lowered by 2–3 °C every week until 18.5–21 °C in the final week. The lighting schedule was 24L:0D on day 1, 23L:1D from day 2 to 7, 20L:4D from day 8 to 11 and 18L:6D from day 12 to 42. The persons taking care of the chickens were masked for the treatments by color-coding the feed bags and pens.

### 2.3. Experimental Protocols

In the completely randomized trial 1, conducted from November–December 2019, newly hatched Ross 308 broilers, 840 in total, were randomly divided into four dietary treatments and each further into seven pens of 30 chickens. Complete formulations adjusted to the demands of fast-growing birds were used (Table 1). The amount of metabolizable energy (ME) and the content of amino acids and minerals in the diets were calculated according to the Nutrient Requirements of Poultry [26]. The diets were produced in a mash form. Premixes for starter and grower feeds contained the chemical coccidiostat diclazuril (Clinacox^R^, Elanco, Greenfield, IN, USA) in the amount of 1 mg/kg of diet. Moreover, all the premixes contained the enzymatic product Rovabio^R^ (xylanase, β-glucanase; Adisseo, Alpharetta, GA, USA) and Phyzyme^R^ XP (phytase; DuPont, Wilmington, DE, USA). The birds received a basal diet (Control) or diets (starter, grower I, II) supplemented with RAC at 125, 250, or 1250 kg/t (RAC125, RAC250, RAC1250, respectively). Selecting the dosing of RAC for was based on the assumption that resin acids are the active ingredient in the product and also in the existing TOFA product (Progres**^®^**; Hankkija Ltd., Hyvinkää, Finland, with 9% resin acids) [9,10,11,12,13]. The recommended TOFA dosing for broiler chickens, 500–1000 g/ton, provides 45–90 g/ton of resin acids. In trial 1 of the present trial, the 125 g/ton dose provided 47 g/ton of resin acids, while the second dose (250 g/ton) provided 94 g/ton of resin acids. The 1250 g/ton level was selected to ensure that moderately exceeding the practical dosing of RAC is tolerated by broiler chickens, and thus, a reasonable safety margin exists for its usage.

For each of the trials 2 and 3, a total of 420 Ross 308 broiler hatchlings were randomly divided into two dietary groups which were divided into 14 replicate pens, 15 birds per pen. These two completely randomized trials were conducted simultaneously from May–June, 2020. Two different complete diet formulations in mash form, supplemented with xylanase, phytase and enzymes and the chemical coccidiostat diclazuril (Clinacox^R^, Elanco, Greenfield, IN, USA) were produced, one for trial 2 and the other for trial 3 (Table 2). The diets were either without RAC (Control) or supplemented with RAC at 175 g/t (RAC175). The 175 g/ton dose was selected in order to ensure that the level of resin acids would be adequate to achieve positive results in many different environmental conditions.

The contents of the basic nutritional components of diets were determined in accordance with the principles of the Weende method [27]. The resin acid concentration of all diets was measured by gas chromatography by a commercial laboratory Oy Separation Research AB (Turku, Finland) by using of GC and GC-MS analysis.

All the three trials lasted for 35 days. During each experiment, the bodyweight of the chickens was monitored at weekly intervals. Feed was weighed at the first day of each week. At the end of each week, the residual feed in feeders was weighed to calculate weekly feed consumption per pen. Dead birds and birds euthanized because of poor health were weighed and the daily mortality was recorded. Mortality was reported as the percentage of birds culled and found dead. Feed conversion ratio (FCR) per pen was calculated by dividing feed consumed (kg) by weight gain (kg) of the respective time period. Feed intake was corrected for mortality by deducting the estimated amount of feed intake of dead birds. Fattening efficiency was determined on the basis of the European Efficiency Index (EEI). To calculate it, the following data were used: length of fattening, feed intake, final bodyweight, and the survival rate in each group:

EEI = (livability × live weight, kg/length of rearing period, days × FCR) × 100.(1)

For each pen, one foot per bird was systematically evaluated for macroscopic examination and scored in three classes of FPD on days 14, 28, and 35 as follows: 0 = No lesions; no or very small superficial lesions, slight discoloration on a limited area, mild hyperkeratosis; 1 = Mild lesion; discoloration of the foot pad, superficial lesions, dark papillae; 2 = Severe lesion; ulcers or scabs, signs of hemorrhages or swollen foot pads according to the classification of Ekstrand et al. [28]. The number of feet in class 0 did not contribute to the score. The FPD score per pen was calculated as follows:

FPD score = [(number of feet in class 1 × 0.5 + number of feet in class 2 × 2) / sample size] × 100 (2)

From all trials, litter quality (moisture) was measured per pen by electronic device (Draminski; Olsztyn, Poland) at days 14, 28, and 35 as a standard procedure at the experimental farm. Measurements of all observations, from five different sites per pen, were averaged per pen.

### 2.4. Statistical Analysis

The pen was considered the experimental unit for statistical purposes. For trial 1, the results were analyzed statistically using one-way analysis of variance (ANOVA) and Duncan’s test. For trials 2 and 3, the results were analyzed statistically using Student’s *t*-test. For both trials, the results were characterized using the arithmetic mean (x), the standard error of the mean (SEM), and the level of significance (*p*). The computer program STATISTICA 12 was used for calculations.

## 3. Results

### 3.1. Feed Analyses

The results of the analysis of nutritional components of the experimental diets are given in Table 1 for trial 1 and in Table 2 for trials 2 and 3. The results of the resin acid analyses of the experimental diets are presented in Table 3 for all the three trials.

### 3.2. Bird Health

In all three trials, the general health of the birds was good during the experimental period, and no medication was used. Birds were growing at typical rate for the breed.

### 3.3. Results of Trial 1

Bird performance results of trial 1 are presented in Table 4. On day 35, the highest body weight was found in groups RAC125 and RAC250. In comparison to the control group, the difference in body weight was approximately 0.12 kg (+4.7%; *p* < 0.01). The final weight of birds in group RAC1250 was similar to weight of control birds. The total feed intake, mortality and FCR were similar in all dietary treatments, although in the second week, RAC at 125 and 250 g/ton tended to improve FCR. The EEI value was significantly improved in the RAC125 and RAC250 groups by 8.1% (*p* < 0.05) and 10.9% (*p* < 0.01), respectively. There was a significant positive effect of RAC addition at 250 and 1250 g/ton on litter quality on days 28 and 35, and for RAC1250, already on day 14 (*p* < 0.05; Table 5). The FPD score was unaffected by the dietary treatments (Table 5).

### 3.4. Results of Trial 2

All results of trial 2 are presented in Table 6. Positive effect of dietary RAC on bird body weight was observed from 3 to 5 weeks of age. On day 35, in comparison with control birds, the difference was approximately 0.07 kg (+3.0%; *p* < 0.05). Feed intake and mortality were similar for both groups. The use of RAC significantly improved EEI (+5.2%, *p* < 0.05). Days 1–35 showed a trend for better FCR in RAC-treated chickens (*p* = 0.067). Litter quality was significantly improved by RAC175, compared with control, on day 14, but similar to control in days 28 and 35. Healthy feet with no FPD lesions were observed in all inspected birds, and thus no differences in the FPD score were found between groups (data not shown).

### 3.5. Results of Trial 3

All results of trial 3 are presented in Table 7. On day 35, RAC-fed birds were on average 0.13 kg heavier than control birds (+6.1%; *p* < 0.01). Feed intake and mortality were not affected by the dietary treatments. For the first four weeks, FCR was similar for both treatments, but for days 1–35, FCR was significantly better for RAC175 group than for the control group (*p* < 0.01). Dietary RAC significantly improved EEI (+10.3%, *p* < 0.01). There was positive effect of RAC175 on litter quality on day 28 (*p* < 0.1) and day 35 (*p* < 0.05). The inspected birds had healthy feet with no FPD lesions, and thus, the treatment groups showed no differences in FPD scoring (data not shown).

## 4. Discussion

The performance and feed efficiency of broiler chickens is closely associated with their intestinal functions [5]. Enteric problems such as dysbiosis and wet litter syndrome have been recognized as risk factors for poor bird performance [22,29], and the associated skin lesions in footpads [29,30] further decrease profitability of broiler production through carcass condemnations [31]. Here, the effects of a novel resin acid-based feed additive RAC on broiler performance, litter quality, and FPD lesions were studied. To the knowledge of the authors, this is the first study in which these parameters have been measured from broiler chickens fed with purified coniferous resin acids.

According to the feed analysis results, the quantity of basic nutritional components in experimental diets were at targeted levels in all three trials. The analysis of resin acids from feed samples confirmed that RAC was dosed to the experimental diets at acceptable accuracy, and none of the control diet samples contained resin acids. The discrepancy between calculated and analyzed resin acid concentrations is mainly due to the feed matrix interfering with the extraction of resin acids, leaving the error of margin of this analysis typically from 10 to 20%, although the possibility of other contributing factors, such as feed mixing and sampling, needs to be kept in mind.

One of the main observations of the present study was the significant increase in the final weight of the chickens by dietary resin acids. On day 35 of all three trials, the weight of birds fed with RAC at 125, 175, or 250 g/ton of feed (which provided 47, 66, or 94 g/ton of resin acids, respectively) was significantly higher than that of control birds, the magnitude of the effect being from 3.0% to 6.1%. The range of body weight improvement in the present study is well in line with two broiler studies, in which dietary TOFA, providing 45 g/ton or 67 g/ton resin acids, increased the final weight of broiler chickens by 3.3% [10] or 3.6% [11], respectively. According to a meta-analysis [32] which studied the effect of prophylactic antibiotics on broiler performance, the daily weight gain of birds for 1–42 days was on average 3.84% higher for birds that had antibiotics in diet compared to control birds (daily weight gain 54 vs. 52 g for birds with or without antibiotics, respectively). Thus, the present study suggests that benefit of bird weight gain over the entire rearing period is similar in magnitude for RAC and antibiotic growth promoters. As soon as prophylactic antibiotic usage ceases to be an allowed or desired option, depending on the local restrictions and market demand, products such as RAC may be seen as valid alternatives to prophylactic antibiotics.

In all three trials, feed intake was similar for all dietary treatments, including the highest RAC dose of 1250 g/ton in trial 1. The result indicates that at doses of 125–1250 g/ton, RAC does not influence the palatability of broiler diets, and that it causes no such physiological effects which would reduce feed intake. In line with this observation, the studies describing the performance effects of TOFA on broiler chickens or turkeys have reported no effects on feed intake [9,10,11,12]. Moreover, the antibiotic growth promoters avilamycin, flavomycin, and virginiamycin do not reduce the feed intake of broiler chickens [32], and in a recent article reviewing mechanisms by which prebiotics improve poultry performance, feed intake is not mentioned as a contributing factor [33].

Mortality was not affected by the dietary treatments. The birds were in normal condition in these trials and not affected by any infections or stress factors which would have increased the mortality over normal levels. The FCR values showed inconsistent results between the trials. Only in trial 3, the resin acid treatment significantly improved FCR for days 1–35, although a trend (*p* < 0.1) towards the same direction was seen in trial 2. The average FCR values of control groups were 1.53, 1.62, and 1.66 for trials 1, 2, and 3, respectively. It can be speculated that the good FCR in trial 1 did not leave much space for improvement. In any case, the ability of RAC to affect FCR in broiler chickens needs to be verified in future studies. For antibiotic growth promoters, an average improvement of FCR is 2.64% [32].

Another way to measure the efficiency of meat poultry production is the EEI, which takes into account broiler mortality, final weight, feed conversion, and duration of the rearing period. In all the three trials of the present study, amending the feeds with RAC at doses of 125–250 g/ton significantly improved EEI by 5.1–10.9%, compared with the respective control groups. The 250 g/ton dose of RAC showed the largest response to EEI. In comparison, TOFA dose providing 45 g of resin acids/ton of feed has been reported to result in 6.4% improvement of EEI [10]. Furthermore, in a 15-week experiment with female turkeys, TOFA amendment providing 90 or 135 g/ton of resin acids improved EEI by 4.1% and 6.0%, respectively [12]. The results suggest that dietary resin acids have potential to improve the efficiency of poultry production.

The motivation to measure litter moisture and footpad lesions arose from a study suggesting that dietary resin acids support gut barrier functions by reducing the inflammation-associated collagen degradation of intestinal mucosa [21]. Gut leakage has been associated with diarrhea [22] which negatively affects litter quality over time. Wet litter has been associated with footpad lesions and hock burns [29,34], and also to poor performance of broilers [22,35]. While there are no prior studies on the effect of pure resin acids on litter moisture, dietary TOFA amendment has been reported to improve litter quality in broiler chickens [10] and turkeys [12]. As the positive effect of good litter quality and broiler performance has been shown [22,35], it may be speculated that the improved litter quality in RAC groups may have contributed to their better growth performance, for example by possibly decreasing pathogen load in the litter. It is noteworthy that in the present study, litter moisture was lower for the RAC1250 group than the other diet groups already on day 14, while the other RAC dosages showed their effect on this parameter on day 28 or 35. Whether the high dose of RAC is especially efficient in reducing intestinal leakage remains to be addressed in future studies.

In contrast to the hypothesis, RAC amendment did not reduce FPD lesions in the present study, although it should be noted that birds in trials 2 and 3 did not suffer from FPD lesions at all. In a previous broiler study, TOFA, providing resin acids 45 g/ton, significantly reduced FPD scores by 18%, but the score of the control group was 91.14 [10]. In 15-week-old female turkeys, a TOFA dose providing resin acids 90 g/ton or 135 g/ton reduced FPD scores by 23% and 28%, respectively, when the average control group score was 114.50 [12].

The 1250 g/ton RAC dosing was designed to give information on safety margins of this dietary additive. The intended level of usage of this feed additive lies between 125 and 250 g/ton, so the 1250 level represents a 5–10 × overdose to this range of levels. As resin acids are fat-soluble molecules not commonly found in feed ingredients, it is necessary to ensure their safety in diets of meat poultry when fed at practical levels. Although published information on the toxicity of resin acids on chickens does not exist, studies on laboratory rodents suggest quite low toxicity, as discussed by Apajalahti et al. [36]. In broiler chickens, resin acids are absorbed from the intestine but secreted via bile back to gut lumen and mostly voided via feces, with levels in blood plasma remaining around 1/1000 of the dietary levels [36]. This indicates an effective clearance of resin acids from the body of broiler chickens. In the present study, even at the 1250 g/ton dosing of RAC for 35 days, no adverse effects were observed on any of the parameters studied here. All the performance parameters measured from RAC1250 were statistically similar to those of other dietary treatments, suggesting that the product is well tolerated by broiler chickens.

## 5. Conclusions

The effects of a novel resin acid-based product, RAC, on broiler chickens were studied in three separate 35-day trials. The positive effects of RAC on broiler performance were demonstrated here for the first time. Higher EEI as a response to dietary resin acids suggests improved profitability of broiler production at the dietary RAC levels of 125–250 g/ton. Resin acid treatments resulted in a reduced litter moisture, but no effects on FPD lesions were observed. The reduced litter moisture may suggest that resin acids support the intestinal condition and thus inhibit the risk for diarrhea. Overall, the results suggest that the resin acid concentrate is a promising feed additive for broiler chickens.

## Figures and Tables

**Table 1:** Composition and nutritional value of basal diets for trial 1.

Item	Trial 1		
	Starter d1–10	Grower 1 d11–21	Grower 2 d22–35
Composition (%):			
Wheat	29.34	36.17	41.39
Maize	25	20	15
Triticale	5	6	8
Soybean meal	29.66	23.89	20.84
Rapeseed meal	4	6	6
Soybean oil	3.10	4.30	5.30
Limestone	1.22	1.2	1.17
Calcium phosphate	1.12	0.88	0.7
Fodder salt	0.23	0.21	0.23
Sodium bicarbonate	0.21	0.16	0.14
L-lysine HCL	0.24	0.29	0.32
DL-Methionine	0.25	0.22	0.23
L-Threonine	0.04	0.08	0.09
Premix *	0.5	0.5	0.5
Nutritional value:			
EM, kcal/kg	2960	3060	3150
Crude protein, %	21.5	20.5	19.5
Lysine, %	1.28	1.2	1.13
Lysine, dig., %	1.16	1.08	1.02
Methionine + cystine, %	0.94	0.89	0.85
Methionine + cystine, dig., %	0.83	0.78	0.75
Threonine, %	0.83	0.80	0.78
Threonine, dig., %	0.71	0.68	0.66
Tryptophan, %	0.25	0.24	0.23
Tryptophan, dig., %	0.22	0.21	0.20
Calcium, %	0.95	0.9	0.85
Available phosphorus, %	0.45	0.4	0.36
Sodium, %	0.18	0.16	0.15
Analyzed chemical composition, %:			
Dry matter	88.38	88.76	89.16
Crude ash	4.72	4.39	4.2
Crude protein	21.44	20.36	19.76
Ether extract	4.55	5.77	6.89
Crude fiber	3.29	3.31	3.53

* Premix composition: Starter—2,600,000 IU vitamin A, 600,000 IU vitamin D3, 8000 mg vitamin E, 600 mg vitamin K3, 400 mg vitamin B1, 1200 mg vitamin B2, 600 mg vitamin B6, 4 mg vitamin B12, 7000 mg nicotinic acid, 2400 mg calcium pantothenate, 200 mg folic acid, 30 mg biotin, 80,000 mg choline chloride, 10,000 mg Fe, 18,000 mg Mn, 3000-mg Cu, 18,000 mg Zn, 200 mg J, 60 mg Se; chemical coccidiostat—diclazuril, enzymatic products, antioxidant, biotin, 400 mg choline chloride, 70.3 mg Fe, 98.8 mg Mn, 15.2 mg Cu, 79.8 mg Zn, 1.9 mg J, 0.23-mg Se; chemical coccidiostat—diclazuril, enzymatic products, antioxidant. Grower 1,2—2,400,000 IU vitamin A, 600,000 IU vitamin D3, 6000 mg vitamin E, 500 mg vitamin K3, 300 mg vitamin B1, 1000 mg vitamin B2, 500 mg vitamin B6, 3 mg vitamin B12, 5000 mg nicotinic acid, 2000 mg calcium pantothenate, 200 mg folic acid, 20 mg biotin, 60,000 mg choline chloride, 20,000 mg Fe, 18,000 mg Mn, 3000-mg Cu, 16,000 mg Zn, 200 mg J, 60 mg Se; chemical coccidiostat—diclazuril, enzymatic products, antioxidant.

**Table 2:** Composition and nutritional value of basal diets for trials 2 and 3.

Item	Trial 2			Trial 3		
	Starter d1–10	Grower 1 d11–21	Grower 2 d22–35	Starter d1–10	Grower 1 d11–21	Grower 2 d22–35
Composition (%):						
Wheat	29.34	36.17	41.39	24.23	29.88	33.08
Maize	25	20	15	25	20	15
Barley	-	-	-	5	6	8
Triticale	5	6	8	5	6	8
Soybean meal	29.66	23.89	20.84	29.66	23.89	20.84
Rapeseed meal	4	6	6	4	6	6
Soybean oil	3.10	4.30	5.30	3.30	4.60	5.70
Limestone	1.22	1.2	1.17	1.22	1.2	1.17
Calcium phosphate	1.12	0.88	0.7	1.12	0.88	0.7
Fodder salt	0.23	0.21	0.23	0.23	0.21	0.23
Sodium bicarbonate	0.21	0.16	0.14	0.21	0.16	0.14
L-lysine HCL	0.24	0.29	0.32	0.24	0.29	0.32
DL-methionine	0.25	0.22	0.23	0.25	0.22	0.23
L-threonine	0.04	0.08	0.09	0.04	0.08	0.09
Premix *	0.5	0.5	0.5	0.5	0.5	0.5
Nutritional value:						
EM, kcal/kg	2960	3060	3150	2960	3060	3150
Crude protein, %	22.5	21.5	20.5	22.5	21.5	20.5
Lysine, %	1.28	1.2	1.13	1.28	1.2	1.13
Lysine, dig., %	1.15	1.08	1.02	1.15	1.08	1.02
Methionine + cystine, %	0.94	0.89	0.85	0.94	0.89	0.85
Methionine + cystine, dig., %	0.83	0.78	0.75	0.83	0.78	0.75
Threonine, %	0.83	0.80	0.78	0.83	0.80	0.78
Threonine, dig., %	0.71	0.68	0.66	0.71	0.68	0.66
Tryptophan, %	0.25	0.24	0.23	0.25	0.24	0.23
Tryptophan, dig., %	0.22	0.21	0.20	0.22	0.21	0.20
Calcium, %	0.95	0.9	0.85	0.95	0.9	0.85
Available phosphorus, %	0.45	0.4	0.36	0.45	0.4	0.36
Sodium, %	0.18	0.16	0.15	0.18	0.16	0.15
Analyzed chemical composition, %:						
Dry matter	89.01	88.57	88.38	88.88	89.2	88.28
Crude ash	4.87	4.14	4.02	4.52	4.23	4.15
Crude protein	22.06	21.66	20.52	22.18	21.54	20.73
Ether extract	4.65	5.75	6.86	5.19	6.21	7.24
Crude fiber	3.22	3.2	3.15	3.22	3.41	3.38

* Premix composition: Starter—2,600,000 IU vitamin A, 600,000 IU vitamin D3, 8000 mg vitamin E, 600 mg vitamin K3, 400 mg vitamin B1, 1200 mg vitamin B2, 600 mg vitamin B6, 4 mg vitamin B12, 7000 mg nicotinic acid, 2400 mg calcium pantothenate, 200 mg folic acid, 30 mg biotin, 80,000 mg choline chloride, 18,000 mg Mn, 18,000 mg Zn, 10,000 mg Fe, 3000-mg Cu, 200 mg J, 60 mg Se; chemical coccidiostat—diclazuril, enzymatic products, antioxidant, biotin, 400 mg choline chloride, 70.3 mg Fe, 98.8 mg Mn, 15.2 mg Cu, 79.8 mg Zn, 1.9 mg J, 0.23-mg Se; chemical coccidiostat —diclazuril, enzymatic products, antioxidant. Grower 1,2—2,400,000 IU vitamin A, 600,000 IU vitamin D3, 6000 mg vitamin E, 500 mg vitamin K3, 300 mg vitamin B1, 1000 mg vitamin B2, 500 mg vitamin B6, 3 mg vitamin B12, 5000 mg nicotinic acid, 2000 mg calcium pantothenate, 200 mg folic acid, 20 mg biotin, 60,000 mg choline chloride, 20,000 mg Fe, 18,000 mg Mn, 3000 mg Cu, 16,000 mg Zn, 200 mg J, 60 mg Se; chemical coccidiostat—diclazuril, enzymatic products, antioxidant.

**Table 3:** Added test ingredient (RAC) and calculated and analyzed values of resin acids in experimental diets.

Item			Resin Acids			
		RAC		Starter	Grower 1	Grower 2
Trial	Diet Groups	Added, g/ton	Calculated, g/ton	Analyzed, g/ton		
	Control	0	0	0	0	0
Trial 1	RAC125	125	47	38	55	42
	RAC250	250	94	87	82	98
	RAC1250	1250	469	379	513	459
Trial 2	Control	0	0	0	0	0
	RAC175	175	66	46	66	71
Trial 3	Control	0	0	0	0	0
	RAC175	175	66	47	72	63

**Table 4:** Production performance of broilers in trial 1.

Item	Diet Groups				SEM	*p*-Value
	Control	RAC125	RAC250	RAC1250		
Bird weight, kg						
Day 1	0.04	0.04	0.04	0.04	0.001	0.137
Day 7	0.17	0.18	0.18	0.17	0.001	0.205
Day 14	0.45 ^ax^	0.48 ^b^	0.47 ^y^	0.47 ^y^	0.004	0.022
Day 21	0.91 ^x^	0.95 ^y^	0.95 ^y^	0.93	0.007	0.072
Day 28	1.57 ^a^	1.65 ^b^	1.63 ^b^	1.57 ^ab^	0.011	0.009
Day 35	2.32 ^a^	2.43 ^b^	2.44 ^b^	2.33 ^ab^	0.016	0.002
Feed intake per pen, kg						
Days 1–7	0.14	0.15	0.15	0.15	0.002	0.168
Days 1–14	0.50	0.52	0.51	0.53	0.005	0.231
Days 1–21	1.14	1.16	1.16	1.17	0.009	0.789
Days 1–28	2.25	2.32	2.30	2.26	0.016	0.104
Days 1–35	3.49	3.61	3.55	3.53	0.023	0.343
Feed conversion ratio						
Days 1–7	1.10	1.10	1.10	1.12	0.008	0.661
Days 1–14	1.23	1.18	1.19	1.23	0.010	0.150
Days 1–21	1.31 ^x^	1.27 ^y^	1.27 ^y^	1.31 ^x^	0.009	0.085
Days 1–28	1.47	1.45	1.45	1.46	0.007	0.714
Days 1–35	1.53	1.51	1.48	1.54	0.010	0.162
Mortality, %	4.76	3.33	2.86	3.33	0.451	0.497
EEI, points	412.4 ^ac^	445.9 ^bx^	457.5 ^ba^	418.7 ^bcy^	5.814	0.008

Different superscripts in same row are significant or trending (a/b: *p* ≤ 0.05; x/y 0.05 < *p* ≤ 0.10). SEM: Standard error of the mean, EEI: European Efficiency Index.

**Table 5:** Litter quality and footpad dermatitis (FPD) score results of trial 1.

Item	Diet Groups				SEM	*p*-Value
	Control	RAC125	RAC250	RAC1250		
Litter quality						
Day 14	15.20 ^a^	15.09 ^a^	14.93 ^a^	12.73 ^b^	0.304	0.004
Day 28	32.33 ^x^	30.84 ^xy^	28.26 ^y^	28.18 ^y^	0.663	0.059
Day 35	56.59 ^a^	54.54 ^ab^	52.99 ^b^	51.63 ^b^	0.534	0.003
FPD score						
Day 14	4.29	4.29	4.29	4.29	0.638	1.000
Day 28	41.43	38.57	36.67	35.71	3.040	0.925
Day 35	63.33	56.19	58.1	55.24	3.233	0.834

Different superscripts in same row are significant or trending (a/b: *p* ≤ 0.05; x/y 0.05 < *p* ≤ 0.10). SEM: standard error of the mean, FPD score: Foot pad dermatitis score.

**Table 6:** Results of Trial 2.

Item	Diet Groups		SEM	*p*-Value
	Control	RAC175		
Bird weight, kg				
Day 1	0.04	0.04	0.001	0.485
Day 7	0.17	0.17	0.001	0.469
Day 14	0.46	0.46	0.003	0.819
Day 21	0.94 ^x^	0.96 ^y^	0.006	0.056
Day 28	1.65	1.67	0.009	0.236
Day 35	2.34 ^a^	2.41 ^b^	0.015	0.017
Feed intake per pen, kg				
Days 1–7	0.17	0.17	0.002	0.393
Days 1–14	0.59	0.58	0.006	0.206
Days 1–21	1.31	1.35	0.012	0.127
Days 1–28	2.50	2.53	0.017	0.420
Days 1–35	3.79	3.83	0.027	0.523
Feed conversion ratio				
Days 1–7	1.02	1.01	0.014	0.666
Days 1–14	1.28	1.25	0.015	0.319
Days 1–21	1.40	1.41	0.013	0.791
Days 1–28	1.52	1.52	0.011	0.871
Days 1–35	1.62 ^x^	1.59 ^y^	0.009	0.067
Mortality, %	3.33	3.33	0.642	1.000
EEI, points	397.6 ^a^	418.2 ^b^	5.076	0.040
Litter quality				
Day 14	15.25 ^a^	14.11 ^b^	0.291	0.049
Day 28	29.58	29.02	0.750	0.720
Day 35	57.69	56.10	0.933	0.405

Different superscripts in same row are significant or trending (a/b: *p* ≤ 0.05; x/y 0.05 < *p* ≤ 0.10). SEM: standard error of the mean, EEI: European Efficiency Index.

**Table 7:** Results of Trial 3.

Item	Diet Groups		SEM	*p*-Value
	Control	RAC175		
Bird weight, kg				
Day 1	0.04	0.04	0.001	0.182
Day 7	0.16	0.17	0.001	0.340
Day 14	0.45	0.46	0.003	0.396
Day 21	0.88	0.88	0.008	0.867
Day 28	1.59	1.61	0.008	0.162
Day 35	2.13 ^a^	2.26 ^b^	0.018	<0.001
Feed intake per pen, kg				
Days 1–7	0.17	0.17	0.002	0.787
Days 1–14	0.59	0.59	0.005	0.794
Days 1–21	1.25	1.24	0.018	0.721
Days 1–28	2.46	2.46	0.017	0.808
Days 1–35	3.54	3.63	0.034	0.179
Feed conversion ratio				
Days 1–7	1.03	1.01	0.010	0.358
Days 1–14	1.30	1.28	0.012	0.359
Days 1–21	1.41	1.40	0.015	0.722
Days 1–28	1.55	1.52	0.012	0.250
Days 1–35	1.66 ^a^	1.61 ^b^	0.011	0.012
Mortality, %	4.29	3.33	0.799	0.561
EEI, points	351.9 ^a^	388.3 ^b^	5.641	<0.001
Litter quality				
Day 14	14.55	13.53	0.394	0.203
Day 28	30.04 ^x^	27.74 ^y^	0.686	0.095
Day 35	59.03 ^a^	54.81 ^b^	0.920	0.019

Different superscripts in same row are significant or trending (a/b: *p* ≤ 0.05; x/y 0.05 < *p* ≤ 0.10). SEM: standard error of the mean, EEI: European Efficiency Index.

## Data Availability

The datasets generated during and/or analyzed during the current study are available from the corresponding author on reasonable request.
